# Inflammation in Vein Graft Disease

**DOI:** 10.3389/fcvm.2018.00003

**Published:** 2018-01-24

**Authors:** Margreet R. de Vries, Paul H. A. Quax

**Affiliations:** ^1^Department of Surgery, Einthoven Laboratory for Experimental Vascular Medicine, Leiden University Medical Center, Leiden, Netherlands

**Keywords:** cardiovascular disease, bypass graft, saphenous vein, vein graft disease, inflammation, innate immunity, atherosclerosis

## Abstract

Bypass surgery is one of the most frequently used strategies to revascularize tissues downstream occlusive atherosclerotic lesions. For venous bypass surgery the great saphenous vein is the most commonly used vessel. Unfortunately, graft efficacy is low due to the development of vascular inflammation, intimal hyperplasia and accelerated atherosclerosis. Moreover, failure of grafts leads to significant adverse outcomes and even mortality. The last couple of decades not much has changed in the treatment of vein graft disease (VGD). However, insight is the cellular and molecular mechanisms of VGD has increased. In this review, we discuss the latest insights on VGD and the role of inflammation in this. We discuss vein graft pathophysiology including hemodynamic changes, the role of vessel wall constitutions and vascular remodeling. We show that profound systemic and local inflammatory responses, including inflammation of the perivascular fat, involve both the innate and adaptive immune system.

## Introduction

**Figure 1:**
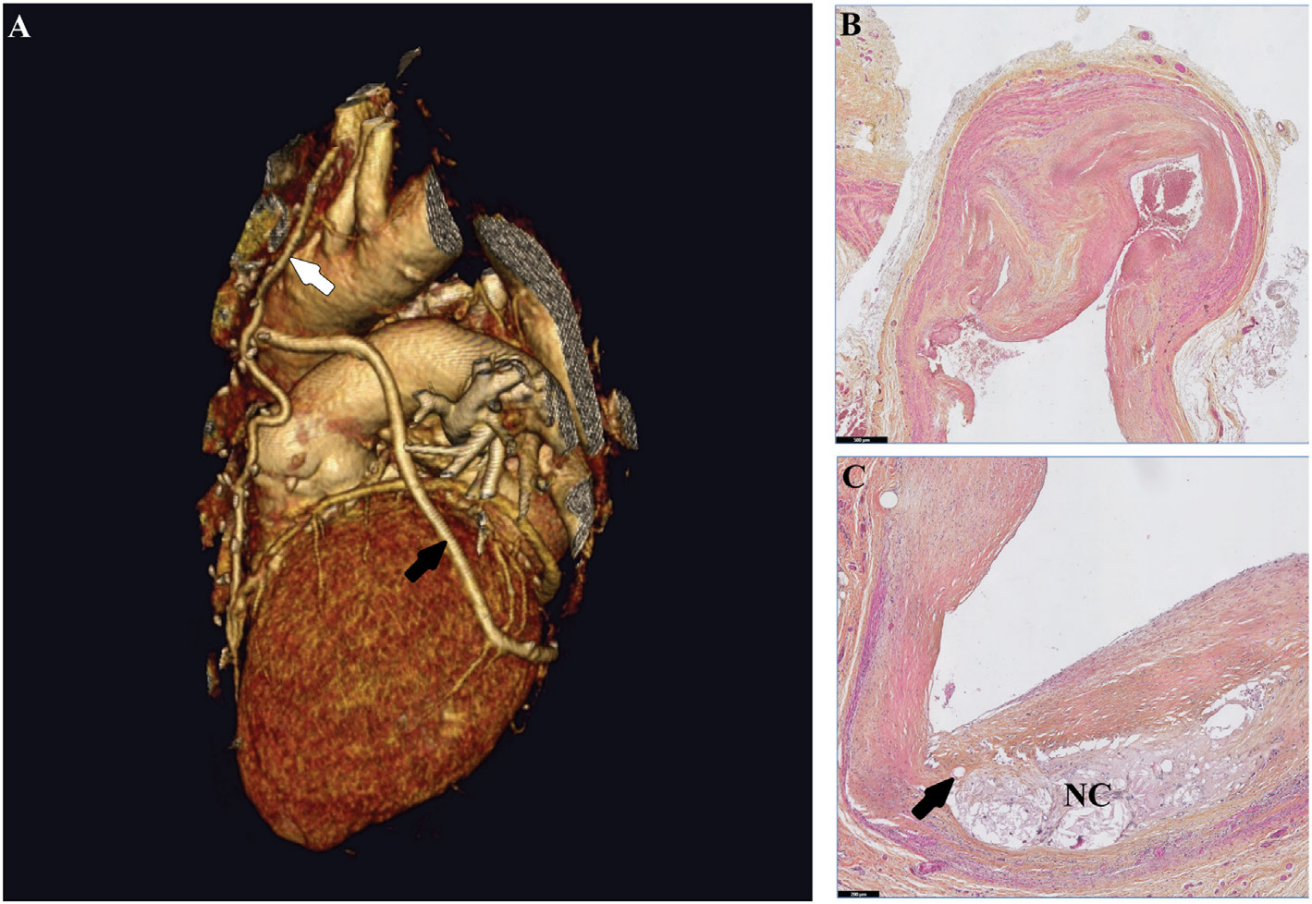
Human vein grafts in macroscopic and microscopic views. **(A)** 3D reconstruction of a heart. In this CT scan, a saphenous vein segment (black arrow) is grafted from the aorta to the ramus circumflexus. The left internal mammarian artery graft (white arrow) is connected to the left anterior descending coronary artery. **(B)** A failed human saphenous vein graft displaying in the intimal hyperplasia, extensive smooth muscle cell accumulation, and extracellular matrix deposition. **(C)** Accelerated atherosclerosis in a human vein graft lesion is characterized by a decellularized necrotic core with cholesterol crystals and calcification (NC) and neovessels (arrow).

Occlusive atherosclerotic disease is a leading cause of mortality and morbidity worldwide. The most commonly used revascularization strategies to unblock or circumvent atherosclerotic lesions are balloon angioplasty (with or without stenting), endarterectomy and bypass surgery. For patients with left main coronary artery disease (CAD), three-vessel CAD and patients with late-stage peripheral artery occlusive disease (PAOD) bypass surgery is the primary standard of care (1–4). For patients receiving a single graft the left internal mammary artery is the graft of choice, since these give the best patency rates (5, 6). However, for bypassing multiple lesions, complex lesions or long diffuse lesions (especially in peripheral artery disease) veins are frequently necessary as a conduit, Figure 1A. Among veins the great saphenous vein is the most obvious conduit and is almost exclusively used as graft in patients with PAOD (7). Advantages of the saphenous vein include the length, which allows the use for multiple grafts, its superficial location for easy accessibility and the expendability (after removal of the vein the surrounding tissue is still perfused by other vessels). Unfortunately, patency rates of vein grafts are poor compared to arterial grafts (1). Due to acute thrombosis patency rates of vein grafts decrease with 10% within the first month (1). Intimal hyperplasia and accelerated atherosclerosis lead to a 40% overall patency after 10–20 years, Figures 1B,C (8, 9). Risk factors associated with vein graft disease (VGD) include age, race, gender, hypercholesterolemia, diabetes mellitus, and chronic kidney disease (10–14). Also factors associated with the surgery contribute to reduced patency. These include the location and quality of the artery where the bypass will be attached, and quality and handling of the venous conduit. Collection of venous conduits with the so called “no touch technique” in which veins are harvested including perivascular fat improve patency rates (15).

**Figure 2:**
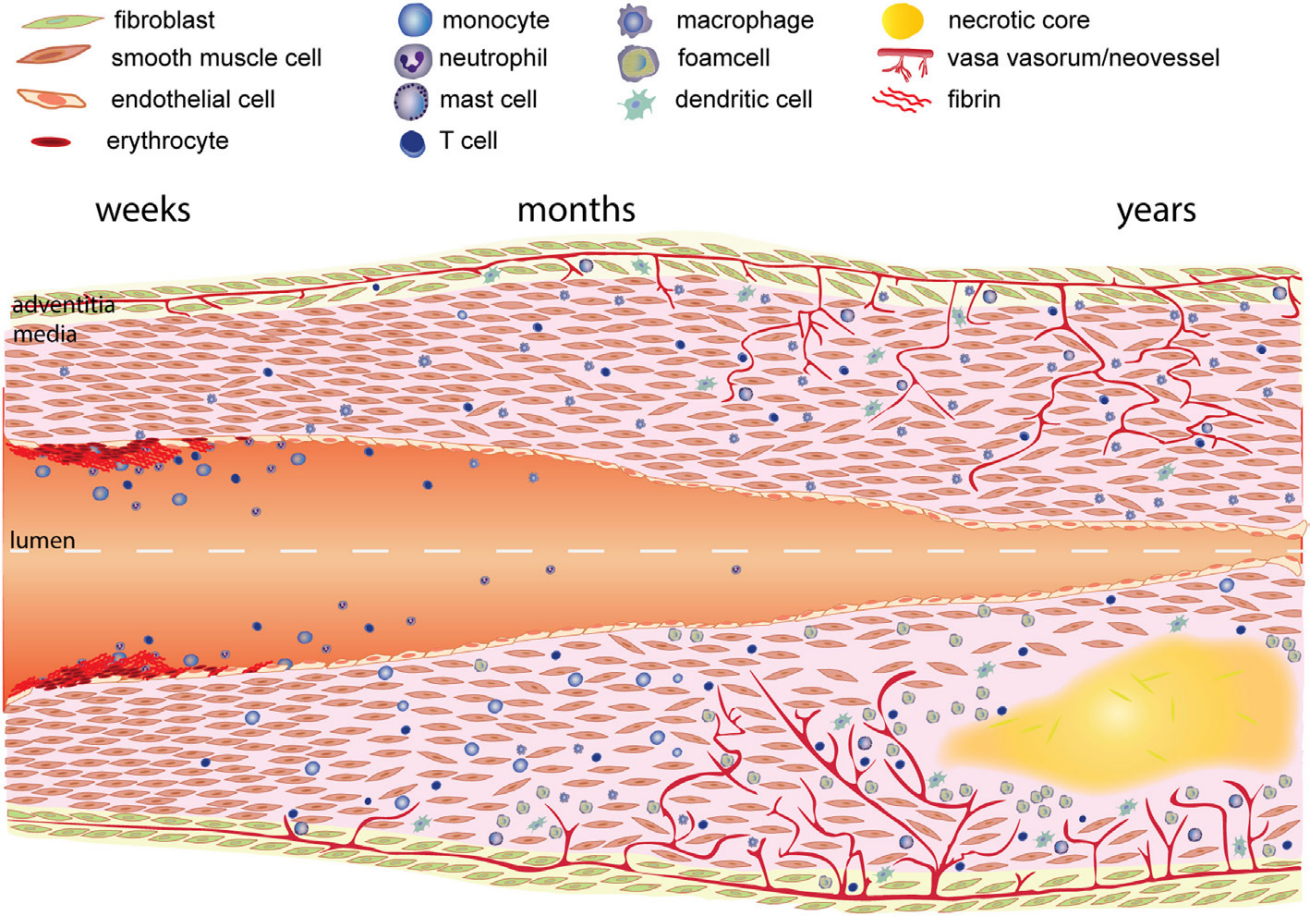
Time course of vein graft development. As a result of the vein graft procedure the endothelial layer is damaged resulting in coverage of the luminal surface by fibrin. White blood cells (neutrophils, monocytes, and lymphocytes) attach and infiltrate the fibrin layer and intima. Next activated smooth muscle cells in the media and fibroblasts in the adventitia are and start migrating toward the intima, forming the intimal hyperplasia. Migration and proliferation of smooth muscle cells is enhanced by growth factors and cytokines released by cells in the vessel wall, and especially inflammatory cells. Growth factors and cytokines also induce extracellular matrix deposition, resulting in further growth of the intimal hyperplasia. The lower part of the figure describes the process of vein graft remodeling as it occurs under atherosclerotic conditions (lower part). Typically macrophages in the vessel wall engulf lipids and become foam cells. Subsequently a necrotic core is formed by dying cells and cholesterol depositions. Hypoxia in the vessel wall induces the growth of plaque neovessels.

In this review, we emphasize the role of inflammatory processes during vein graft remodeling and show how inflammation is involved in all phases leading to VGD, Figure 2. Currently, statins and aspirin are the only treatment options recommended for both CAD and PAOD patients (4, 16–18). Although a lot of research is performed on new targets and therapies it is somewhat disappointing that no effective new strategies that prevent VGD have come up. The recent published results of IL1β inhibition with canakinumab resulting in positive effects on atherosclerosis (19) are very encouraging for new studies targeting inflammation in VGD. In this review, we discuss the pathophysiology of vein grafts and the role of inflammatory mediators during this process based on preclinical and clinical research.

## Vein Graft Remodeling

Remodeling of the vessel wall of the vein grafts is a crucial process during all subsequent stages of VGD. The initial remodeling event is the distension of the venous segment during surgical harvesting and subsequent controlling for proper ligation of all side branches. Usually this is done by checking the lack of leakage of fluids *via* these side branches when pressure is inflicted on the isolated venous segment, leading to a profound distension of the venous segment. In the next stage, directly after grafting the venous segment in the arterial circulation, the vein graft will be distended again due to the exposure to the arterial blood pressure. Both forms of distension lead to serious damage of the vessel wall (20). Not only endothelial cells become damaged or activated but also the media becomes activated due the distension injury, leading to activation of smooth muscle cells (SMCs) and degradation of several components of the extracellular matrix (ECM) in the media as well as the adventitia. These degradation products of matrix elements like hyaluronic acid, proteoglycans and fibronectin are damage-associated molecular patterns (DAMPs), which can act as endogenous ligands for toll-like receptors (TLRs), thus triggering an initial inflammatory response in vein graft remodeling. Moreover, the ischemia-reperfusion period during and after surgery can lead to generation of DAMPs and as well as reactive oxygen species, resulting in damage of vascular cells and upregulation of cytokines (1). Within the first days to weeks this results in influx of inflammatory cells in the vein graft vessel.

**Figure 3:**
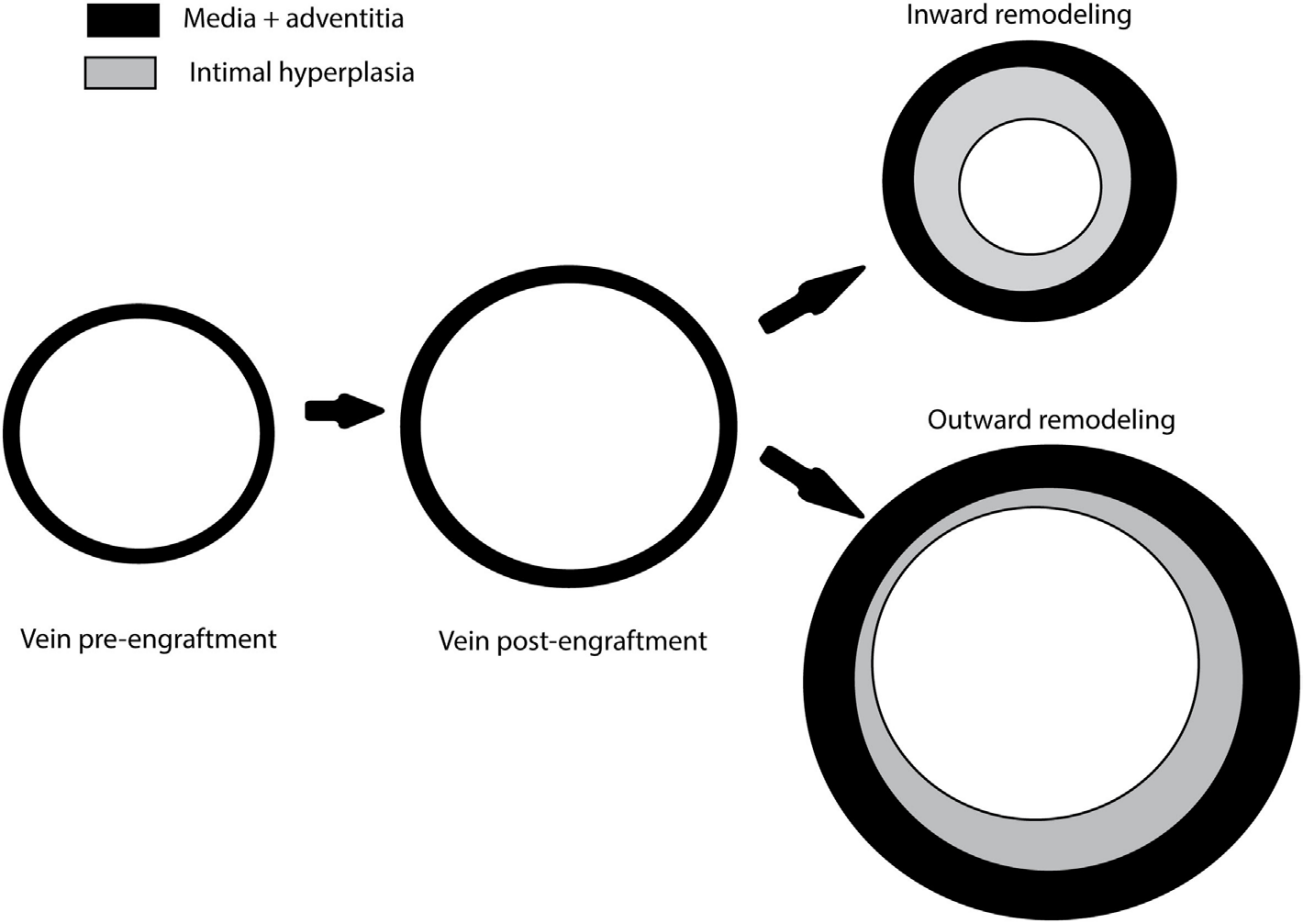
Vein graft remodeling. Damage caused by graft handling and distension during the high-pressure check for leakage as well as implantation in the high blood pressure surrounding of the arterial circulation results in distention of the venous graft. Depending on local and systemic influences like inflammatory factors, this can result in inward remodeling characterized by intimal hyperplasia and a reduced lumen or outward remodeling characterized by moderate intimal hyperplasia and an increased lumen.

The next step in vein graft remodeling relates to the adaptation of the venous segment to the arterial blood pressure. In the media an arterialization process is initiated based on the proliferation of SMCs. This initially beneficial vascular remodeling process, however, may result in an uncontrolled proliferation and migration of SMCs and even myofibroblasts originating from the adventitia and triggers intimal hyperplasia (1). The concomitant outward remodeling of the vein grafts usually compensates for the pathological lumen loss. However, outward remodeling does not occurs always, resulting in situations in which neointima formation leads to inward remodeling as a result of pathological intimal hyperplasia and lumen loss, Figure 3 (21, 22). This is often accompanied or even enhanced by infiltration of inflammatory cells, mainly macrophages, into the vein graft wall (23). Moreover, in the later stage of vein graft remodeling, under hypercholesterolemic conditions, uptake of lipids cause macrophages to turn into foam cells. Macrophage apoptosis leading to necrotic core formation and intraplaque hemorrhage further accelerates the process of VGD by formation of atherosclerotic lesions with an unstable phenotype (1). These accelerated atherosclerotic lesions in the vein grafts represent an end stage in vein graft remodeling which cause long-term (>2 years) vein graft failure (8, 24).

## Contribution of Vessel Wall Constitutions to VGD

### Endothelial Cells

One of the first critical events that a vein has to withstand is a period of ischemia followed by reperfusion during and directly after surgery. In addition graft handling also causes damage to the grafts as well as distension that occurs during the high-pressure check for leakage (1). This leads to increased oxidative stress and cytotoxic activation, which on its turn results in endothelial cell loss (25, 26). Remaining endothelial cells can become apoptotic, damaged, or activated, as shown by expression of ICAM 1, VCAM1, and selectins (27–29). Damaged endothelium shows impaired vasorelaxation as a result of reduced endothelial nitric oxide synthase and NO production (30). The increase in oxidative stress and damage to the endothelium is in particularly seen in vein grafts compared to arterial graft (31, 32).

Following endothelial denudation, the ECM components underneath the endothelium such as collagen, elastin, and proteoglycans become exposed and can trigger coagulation processes leading to fibrin deposition on the luminal surface (33). Fibrin formation and fibrin resolution is tightly regulated by thrombosis and fibrinolysis.

Re-endothelialization begins rapidly after the initial damage. Proliferating endothelial cells are observed within the first week after vein graft surgery in experimental models resulting in a nearly intact endothelial lining 4 weeks after the surgery (27, 28, 33, 34). The duration of the re-endothelialization process in humans is not exactly known but it is likely that this takes somewhat more time. The endothelium-dependent relaxation as observed in human vein grafts, indicates that the grafts have seemingly functional endothelial cells (35). It is unknown whether graft endothelial cells in humans originate from the graft, the adjacent arterial tissue or from the circulation progenitor cells or a combination thereof. In a murine vein graft model it was shown that endothelial cell originate primarily from host vasculature instead of the donor vein (36). Interestingly, in humans allografts both host derived and donor derived endothelial cells were found (37).

Both circulating and local (adventitial) progenitor cells have been shown to contribute to re-endothelialization (38–40). Inducible nitric oxide synthase enhances endothelial progenitor cell attachment and differentiation (41). Homing of these progenitor cells is directed by inflammatory-type macrophages and is most probably integrin β3 dependent (42, 43).

Despite the fact that enhancement of re-endothelialization is beneficial in preventing VGD in preclinical studies, no effective therapeutic approaches exist to facilitate this process (1). Therefore, a potential future therapeutic target in which the inflammatory reaction may play a role could be the promotion of endothelial progenitor cell homing to the damaged endothelium in the grafts.

### Smooth Muscle Cells

Proliferation and migration of SMCs are key elements in intimal hyperplasia formation. During harvesting and engraftment, SMCs within the vein graft are exposed to ischemia resulting in SMC apoptosis (25, 44, 45). Remaining SMCs can change from a quiescent contractile phenotype to a dedifferentiated, proliferating synthetic phenotype. These cells can migrate from the media to the intima of the graft. Alternatively SMCs may migrate from the anastomosed artery toward the intima of the graft (46, 47). Both arterial and venous SMC have been shown to contribute to the intimal hyperplasia in vein grafts (48, 49). Interestingly, after engrafting of a venous segment in the arterial circulation venous marker Ephrin B4 was decreased pointing toward a loss of venous identity during arterialization (50).

Smooth muscle cells in vein grafts express different growth factors such as PDGF, TGF-β, vascular endothelial growth factor, and endothelin-1, which are major stimulators of intimal hyperplasia formation (51–54). Targeting of growth factors or their receptors in preclinical models interfere with this intimal growth (55–58). Arterial and venous grafts display a different pattern of expression of growth factors and signal transduction pathway factors (45, 48, 59), which might contribute to the lower patency rates of venous grafts. Venous SMCs show enhanced MAPK dependent proliferation in comparison to arterial SMCs (60). SMCs and especially activated SMCs produce cytokines such as tumor necrosis factor alpha (TNFα) and C-C motif chemokine CCL2 (also known as MCP-1) (61, 62), which can increase the Rho/Rac GTPase signaling cascade leading to enhanced SMC migration and proliferation (63).

Veins possess elastin fibers but lack defined external and internal elastic lamina. Therefore, fibroblasts when migrating from the adventitia to the intima in veins encounter little barriers. These adventitial fibroblasts are highly proliferative. Adventitial fibroblasts that acquire a smooth muscle-like phenotype are known to contribute to intimal hyperplasia formation (64, 65).

Adventitial and bone marrow-derived progenitor cells also contribute to the thickening of the vein graft wall (38, 39, 66, 67). Already during vein graft surgery the bone marrow releases functional active CXCR4^+^ progenitor cells (68). Although a substantial portion of the bone marrow-derived cells express a SMC phenotype, they do not fully acquire the complete SMC lineage phenotype (69). Cytokine dependent activation of Stem Cell Antigen-1^+^ positive bone marrow-derived progenitor cells results in enhanced SMC migration and proliferation (70). Vein grafting in mice deficient in CXCR4 resulted in reduced vein graft thickening (71). Also knock down of fibroblast-specific protein-1 in bone marrow cells inhibited intimal hyperplasia (72).

### Extracellular Matrix

In the initial phase after vein graft surgery exposure of components of the ECM interact with plasma components and platelets and contribute to the thrombogenic luminal surface of the vein graft. ECM components such as fibronectin, heperansulphate and other proteoglycans can act as DAMPs which interact with pattern recognition receptors like TLRs, thereby initiating a proinflammatory response, primarily directed by nuclear factor kappa-light-chain-enhancer of activated B cells (NF-κB) (73). The ECM component hyaluronic acid is especially found in arterialized vein grafts and contributes to vein graft intimal thickening by inducing SMC proliferation (74). In the first phases of vein graft remodeling, upregulation of TGF-β is linked to increased mRNA expression of collagen I, and collagen III (75, 76). In a rabbit vein graft model late stage remodeling (>1 month) enhanced TGF-β expression was observed which was accompanied by increased fibrosis and reduced matrix metalloproteinase (MMP) 2 and MMP9 activity (77). In rats inhibition of TGF-β signaling resulted in reduced intimal hyperplasia as a result of increased MMP activity (58). The proteases that degrade collagen and other components of the ECM are highly expressed in vein grafts, especially MMP2 and MMP are extensively researched (78–81). MMPs can be secreted by both macrophages and SMC in the vessel wall, however, most interactions with the MMP system results in effects on SMC migration and proliferation and ECM build up. Gene therapeutic approaches in saphenous vein SMCs to silence expression of MMP2 and MMP9 demonstrated reduced SMC migration through a matrigel barrier (82). Moreover, in MMP9 knockout mice the lesion composition was changed due to an increase in collagen content while intimal hyperplasia was similar as in control mice after vein grafting (83). Inhibition of MMPs by the general MMP inhibitor doxycycline resulted in decreased intimal hyperplasia formation in murine vein grafts (80). Overexpression of tissue inhibitors of MMPs (TIMP) 1, 2, and 3 in vein graft models in various experimental animals resulted in intimal hyperplasia formation due to reduced SMC migration and proliferation and inhibition of MMP activity as well as reduced infiltration and migration of inflammatory cells (84–88).

Plasmin, formed by activation of plasminogen by plasminogen activators, can contribute to activation of MMPs and can cleave ECM components, such as laminin and fibronectin. These ECM degradation products subsequently can bind to pattern recognition receptors (89). Failed human vein grafts demonstrated an increased expression of members of the plasminogen activation system (90, 91). The plasminogen activation system consists of two main PAs, urokinase-type PA (uPA) and tissue-type PA (tPA). uPA is essential in extracellular proteolysis, cell migration, and matrix remodeling, while tPA is mainly involved in fibrinolysis (92). In porcine vein grafts overexpression of tPA reduced early vein graft thrombosis (93). Adenovirus mediated overexpression in human saphenous explants of a hybrid protein consisting of the receptor-binding amino terminal fragment (ATF) of urokinase and bovine pancreas trypsin inhibitor (BPTI) was able to potently reduce intimal hyperplasia formation (94). A hybrid protein consisting of TIMP1 and the aforementioned ATF was constructed to inhibit MMP activity locally at the cell surface (95). This construct reduced vein graft thickening and preserved the luminal area (96). A third protein was constructed by combining the three constructs resulting in TIMP1.ATF.BPTI that was capable of inhibiting both plasmin and MMP activity at the cell surface, which effectively reduced vein graft intimal hyperplasia and outward remodeling (97). Plasminogen activator inhibitor 1 (PAI1), another plasminogen activator, showed also clear effects on vein graft remodeling. PAI1-deficient mice showed enhanced intimal hyperplasia due to increased thrombin activity (98).

## Inflammation

The immune system plays an important role during all phases in vein graft development (99, 100). Immediately after surgery DAMPs are released which *via* binding to the TLRs activate the cells in the vessel wall resulting in the release of growth factors and cytokines. DAMPS can also activate platelets and thus enhance, due to the platelet expressed adhesion molecules P- and E-selectin, the attachment of circulating leukocytes and subsequent infiltration into the vessel wall (101). Neutrophils are mainly detected on the de-endothelialized lumen within the fibrin layer that is formed there (102). Neutrophils are phagocytes that also produce MMP2 and MMP9, as well as other proteases and a wide array of growth factors with which they can influence neighboring cells in the vessel wall (103). Monocytes enter the vein graft *via* adhesion to the luminal surface or *via* neovessels in the adventitia (104). Macrophage colony stimulating factor turns these invasive monocytes into macrophages. Macrophages represent the vast majority of inflammatory cells in the vein graft wall and by producing and releasing various cytokines and growth factors influence intimal hyperplasia formation (105). Direct or indirect inhibition of macrophages, by targeting macrophage activating factors have been shown to be successful strategies in preventing the inflammatory response and VGD (61, 106, 107). In addition, various types of inflammatory cells seem to be derived from the adventitia, which consists of loose connective tissue, small neovessels, and nerves. Dendritic cells, mast cells, natural killer (NK) cells, T, and B cells are primarily found in the adventitia (108).

The late phase of vein graft development is characterized by oxidized low-density lipoprotein (ox-LDL) retention and subsequent lipid accumulation (24, 34). Phosphorylcholine is one of the neoantigens exposed by LDL oxidation that can elicit an immune response. Passive immunization with anti-phosphorylcholine antibodies resulted in a reduced inflammatory phenotype which prevents vein graft atherosclerosis in a hypercholesterolemic murine model (109). Interestingly, low levels of natural antibodies against phosphorylcholine in humans are associated with VGD (110). Phosphorylcholine is one of the many ligands for TLRs, like the DAMPs that are expressed upon damage to the vein graft wall. TLRs are central in the induction of inflammatory responses in vascular cell types, and can activate inflammatory cells of both the innate and adaptive immune system (1, 73, 111). TLR activation generally lead *via* the myeloid differentiation primary response protein 88 (MyD88) pathway, to activation of NF-κB and results in induction of cytokines (112). In general, proinflammatory cytokines stimulate vein graft remodeling (99, 113–115). These cytokines activate inflammatory cells but also stimulate SMC migration and proliferation as well as activation of endothelial cells.

NF-κB is one of the most important transcription factors for promotion of inflammatory responses in vein graft remodeling (1). Inhibition of NF-κB resulted in reduced inflammatory responses and attenuation of vein graft thickening in experimental models (116–118). The downstream NF-κB targets CCL2 and TNFα both have been linked to VGD (119, 120). Overexpression of a dominant negative form of CCL2 reduced accelerated atherosclerosis and monocyte invasion in vein grafts in mice on a hypercholesterolemic diet (61). Furthermore, lentivirus mediated overexpression of a shRNA silencing the CCL2 receptor, CCR2, inhibited SMC proliferation and migration and reduced vein graft thickening (121). TNFα is one of the early up-regulated factors in vein graft development. This induction is thought to be a result of the early activation of TLRs. In TNF receptor-1-deficient mice, reduced CCL2 expression and SMC proliferation resulted in reduced vein graft intimal hyperplasia (122). Furthermore, TNF receptor-2-deficient mice also showed reduced vein graft thickening as a result of endothelial cell apoptosis (123). An overview of the various inflammatory factors that are linked to VGD is given in Table 1.

**Table 1:** Inflammatory factors involved in vein graft disease (VGD).

Target/treatment	Effect on VGD	Experimental animal	Reference
Notch ligand delta-like 4	+	Mouse	Koga et al. (107)
Dexamethasone	−	Mouse	Schepers et al. (124)
Annexin A5	−	Mouse	Ewing et al. (125)
Phosphorylcholine antibodies	−	Mouse	Faria-Neto et al. (109)
Il1	+	Mouse	Yu et al. (115)
NF-κB	+	Dog	Shintani et al. (116)
NF-κB	+	Rabbit	Miyake et al. (117)
NF-κB	+	Rat	Meng et al. (118)
MCP-1/CCL2	+	Dog	Tatewaki et al. (119)
MCP-1/CCL2	+	Mouse	Fu et al. (120)
MCP-1/CCL2	+	Mouse	Schepers et al. (61)
CCR2	+	Mouse	Eefting et al. (121)
TNF-R1	+	Mouse	Zhang et al. (122)
TNF-R2	−	Mouse	Zhang et al. (123)
TLR4	+	Mouse	Karper et al. (73)
TLR4	+	Mouse	Nguyen et al. (126)
RP105	+	Mouse	Wezel et al. (127)
C1 inhibitor	−	Mouse	Krijnen et al. (128)
C3	+	Mouse	Schepers et al. (129)
C5a	+	Mouse	de Vries et al. (130)
C5a	+	Mouse	Wezel et al. (131)
Mast cell	+	Mouse	de Vries et al. (130)
Mast cell	+	Mouse	Wu et al. (132)
Natural killer cells	+	Mouse	de Vries et al. (133)
Interferon regulating factor 3 and 7	−	Mouse	Simons et al. (134)

## Immune Modulation

### Toll-Like Receptors

As indicated above, TLRs play a crucial role in the early inflammatory triggers that initiate vein graft remodeling. Among the first mediators of inflammation in vein grafts are DAMPs such as aggrecan and heat shock proteins (135, 136). Endogenous DAMPs activate TLRs that are expressed by cells in the vein graft wall such as endothelial cells and SMCs (1, 73, 137). Exaggerating this response by applying low dose lipopolysaccharide, a strong TLR4 ligand, topically on the vein graft resulted in a strong induction of the inflammatory response and increased intimal thickening (126). Blocking TLR4 in a murine vein graft model, either by genetic deletion or by lentiviral mediated local shRNA silencing, reduced outward remodeling and intimal hyperplasia formation, due to the suppressed inflammatory responses (73). Ligation of the carotid artery in TLR4-deficient mice showed outward remodeling without intimal hyperplasia formation in the non-ligated artery (111). It is therefore suggested that TLR4 affects hemodynamic adaptations and vascular remodeling independently of intimal hyperplasia formation (1). Inhibition of the TLR4 homolog radioprotective 105 aggravated intimal hyperplasia formation in vein graft by increased proinflammatory macrophage proliferation and enhanced SMC migration and proliferation (127). Comparable results were found in vascular remodeling models for restenosis and arteriovenous fistula (138, 139). Whereas in atherosclerosis models a reduction of atherosclerosis could be observed due to the specific function of RP105 on B cells and inhibition of CCR2 dependent macrophage migration (140, 141). Next to the role of TLRs, other components of the innate immune system such as members of the complement cascade are linked to vein graft remodeling.

### Complement System

The complement cascade is a large family of acute response effector and regulatory proteins that is a prominent member of the innate immunity. Vein graft surgery activates the complement system and continues during the vein graft remodeling process since complement factors are present and produced locally in the vein graft wall (129). Inhibition of the key complement factor C3 resulted in reduced intimal hyperplasia by reducing inflammatory cell influx in murine vein grafts (129). C1inh a natural occurring protease inhibitor of the serpin family and alternative pathway component prevented endothelial cell damage in *ex vivo* perfused human saphenous vein segments and reduced vein graft intimal hyperplasia in a murine model (128). In the same *in vitro* perfusion model it was shown that the endogenous complement inhibitor, the C4b-binding protein, was present in the saphenous vein wall and has protective mechanisms to cellular stress and inflammation (142). C5a is one of the major biologically active components of the complement cascade downstream of C3 and exerts its function including chemotaxis of monocytes and mast cells mainly *via* the canonical C5a receptor. Local application of C5a on the vein graft resulted in increased intimal hyperplasia in a mast cell dependent manner (130). Furthermore, acute application of C5a results in enhancement of plaque disruption (131). Inhibition of complement factors seems to be a very promising strategy for preventing VGD in humans. Most interestingly, the mortality in high-risk surgical patients undergoing CABG surgery was reduced by intravenous administration of an antibody against complement factor C5 (pexelizumab) (143).

### Mast Cells

Mast cells are large granular cells that upon activation by IgE, cytokines (TNFα, IL1) and complement factors release granules containing tryptase, chymase, and histamine (144). Vein grafts are rapidly repopulated with mast cells; it should be noted that resting as well as activated mast cells can be found mainly in the perivascular region of vein grafts but not so much in the vessel wall itself (130, 132, 145). Mast cell-deficient mice showed a reduction in intimal hyperplasia in vein grafts, as well as a general reduction of vascular inflammation (130, 132). Moreover, activation of mast cells locally resulted in more unstable lesions and features of plaque rupture (130). The strong effect of mast cells on lesion instability is also seen in native atherosclerosis (146). Remarkably, in these lesions, most mast cells were found in the close vicinity of plaque neovascularization (146).

### NK Cells

Also present in the perivascular region of vein grafts and especially in the adventitia are NK cells (133). Upon activation NK cells secrete lytic granules containing perforin and granzymes and various proinflammatory cytokines (147). The NK cell function is reduced in BALB/C mice due to the lack of crucial NK cell genes of the Ly49 receptor family. When vein graft surgery was performed in BALB/c mice congenic for the C57BL/6 NK gene region, these mice displayed a similar degree of intimal hyperplasia as C57BL/6 mice, while BALB/c mice showed significantly less vein graft remodeling and intimal hyperplasia (133). Furthermore, a decrease in inflammatory cells and interferon-γ expression in the vein graft wall was observed.

### Dendritic Cells

Dendritic cells, originating from Ly-6C^lo^ monocytes, are found in all layers of the vein graft and colocalize with T cells as antigen presenting cells (148). In vein grafts dendritic cells are capable of triggering T cells by costimulation of CD40 (149).

The involvement of adaptive immunity members in VGD is less established than the role of the innate immunity. The participation of the adaptive immune system in vascular diseases is clear and the role in atherosclerosis and restenosis is well described (150).

### T and B Cells

T and B cells have been identified in vein graft lesions, however, no further characterization of subtypes are performed (37, 151). It has been shown that T cells are capable of proliferation in vein grafts (152). Furthermore, interaction between dendritic cells and T cells in a CD40 dependent manner have been observed in vein grafts (149). However, little is known about the exact function and role of T cells in the pathophysiology of VGD. In a recent study, we demonstrated that downstream TLR signaling *via* interferon regulatory factor (IRF) 3 and 7 results in a protective effect on vein graft remodeling. This is particularly of interest since IRF3 and IRF7 activation leads to expression of type1 interferons, that are subsequently involved in the activation of CD4 and CD8^+^ T cells (134). Further studies to investigate the role of (subtypes) of T as well as B cells in VGD are definitely needed.

## Perivascular Adipose Tissue (PVAT)

Most blood vessels, including the saphenous vein, are surrounded by PVAT. In the last decades the vasoactive role of PVAT and adipokines derived from PVAT on vascular function are more and more appreciated (153). PVAT harbors numerous amounts of inflammatory cells. Damage to PVAT results in an inflammatory response driven by adipocyte-derived factors such as resistin, leptin, or the cytokines IL-6, TNF-α, and CCL2 (154). Protective effects of adiponectin on NADPH oxidase, superoxide production and NO bioavailability in the vessel wall are reduced after PVAT damage (155). The “no touch” technique of saphenous vein harvesting is in part based on the beneficial effects of preservation of PVAT and PVAT derived leptin (155, 156). Interestingly, PVAT surrounding different blood vessels differs in its response to injury. Different responses are found between PVAT surrounding saphenous veins and internal mammary arteries (157) but also between internal mammary arteries and coronary arteries (158), pointing to a cause of the encouraging patency rates of internal mammary arteries.

## Accelerated Atherosclerosis and Late Stage Failure

Comparable to native atherosclerosis, hypercholesterolemia, an import driver of VGD and lipid burden, is clearly associated with vein graft age (159). Analysis of human vein grafts obtained at autopsy has shown that coronary vein grafts undergo rapid atherosclerotic lesion development (24). Lesions in coronary vein graft differ from native lesions in having a more concentric and diffuse appearance. Furthermore, the tendency to rupture and occlude due to thrombosis is very high in these vein grafts (8). Especially, older vein grafts (>2 years) fail most frequently due to accelerated atherosclerosis and rupture of lesions (8, 160, 161). Coronary bypass graft occlusion is clearly associated with presence of necrotic core, calcification and negative remodeling (162). Peripheral vein grafts probably suffer less from accelerated atherosclerosis, since these lesions mostly consist of SMCs (163). Occlusion of peripheral vein grafts is frequently linked to high rates of circulating inflammatory cells (100). Circulating inflammatory cells are now being studied as predictors for VGD; both ratios of platelet-monocyte reactivity or lymphocytes-to-monocytes ratios show correlations with VGD (100, 164).

Foam cell formation is already observed in one year old vein grafts. This is followed by necrotic core development between 2 and 5 years after surgery. Intraplaque hemorrhage, most likely originating from leaky angiogenic neovessels in the lesion is also observed in these advanced lesions (24). Plaque angiogenesis and intraplaque hemorrhage are important causes of plaque destabilization and rupture (165). In vein grafts in hypercholesterolemic mice various features linked to late phase graft failure are observed, including angiogenic neovessels, intraplaque hemorrhage, necrotic cores and rupture (1, 88). Especially the presence of plaque neovessels and intraplaque hemorrhage in this model are interesting, since this is a rare observation in atherosclerotic experimental murine models. Improved lesion stability and decreased plaque rupture could be achieved by up regulation of the MMP inhibitor TIMP-1 (88). Targeting inflammatory factors such as annexin A5, mast cells, complement factors and TLRs are effective strategies to not only inhibit intimal hyperplasia formation and accelerated atherosclerosis but also to alter plaque composition and reduce plaque rupture (125, 127, 130, 131).

## Clinical Pharmacological and Surgical Interventional Strategies

Platelet activation and thrombin production are key triggers of early vein graft failure. Antiplatelet therapy starting directly after surgery to prevent early vein graft thrombosis is recommended for both CAD and PAOD patients. Aspirin treatment alone or dual antiplatelet (aspirin and clopidogrel) treatment have been shown to be effective in preventing graft occlusion (166, 167). In both Europe and USA, antiplatelet therapy is recommended to be continued until at least 3 months after the surgery and in some cases indefinitely (4, 17).

Comparable to native atherosclerosis statins are included in the standard of care for patients undergoing vein graft surgery. The mode of actions of statins is primarily cholesterol lowering by inhibiting HMG-CoA reductase but other mechanisms are also described. Statins can improve endothelial function, prevent proliferation of SMCs and decrease activation of macrophages (168, 169). Statin therapy has been proven to prevent vein graft stenosis in both coronary and peripheral grafts (170–172).

A new therapy to prevent VGD is the use of an extravascular support. The extravascular support functions as a protective outer layer of the vein graft, thereby reducing wall tension, activation and stretching of SMCs and endothelial cells (173). Promising results are obtained in *in vitro* and experimental studies (174–177). The recently reported positive preliminary clinical results from a study by Ferrari et al. using an external mesh demonstrate the possibility to improve long-term graft durability (178). The VEST trial showed an improvement in lumen uniformity after external stenting 1 year after CABG surgery in comparison to non-stented vein grafts in the same patients (173, 179, 180). Further elaboration on these studies is needed to solidify the concept of extravascular support on graft patency.

## Conclusion

The use of vein grafts as a revascularization strategy is still necessary despite the unfavorable patency outcomes. Constrictive remodeling, intimal hyperplasia formation, and unstable atherosclerotic lesions are the main causes of VGD in both coronary and peripheral vein grafts. Histopathological studies of human vein grafts and experimental vein graft models have demonstrated that inflammatory components, especially those from the innate immune system, are crucial in all stages of vein graft development. Additional studies are required to prevent VGD and test new strategies for the treatment of vein grafts. Targeting inflammation either in a broad form or in a very specific has great potential as revascularization strategy for failing grafts.

## Author Contributions

MV and PQ designed and wrote the manuscript.

## Conflict of Interest Statement

The authors declare that the research was conducted in the absence of any commercial or financial relationships that could be construed as a potential conflict of interest.
